# A hierarchical spatial modelling approach to investigate MRSA transmission in a tertiary hospital

**DOI:** 10.1186/1471-2334-13-449

**Published:** 2013-09-30

**Authors:** Fiona Kong, David L Paterson, Michael Whitby, Michael Coory, Archie CA Clements

**Affiliations:** 1 Infectious Disease Epidemiology Unit, School of Population Health, University of Queensland, Queensland, Australia; 2 Center for Healthcare Related Infection Surveillance and Prevention, Queensland Health, Queensland, Australia; 3 Centre for Clinical Research, University of Queensland, Queensland, Australia; 4 Greenslopes Clinical School, University of Queensland, Queensland, Australia

**Keywords:** MRSA, *Staphylococcus aureus*, Spatial model, Spatial clustering

## Abstract

**Background:**

Most hospitals have a hierarchical design with beds positioned within cubicles and cubicles positioned within wards. Transmission of MRSA may be facilitated by patient proximity and thus the spatial arrangements of beds, cubicles and wards could be important in understanding MRSA transmission risk. Identifying high-risk areas of transmission may be useful in the design of more effective, targeted MRSA interventions.

**Methods:**

Retrospective data on numbers of multi-resistant and non-multiresistant MRSA acquisitions were collected for 52 weeks in 2007 in a tertiary hospital in Brisbane, Australia. A hierarchical Bayesian spatio-temporal modelling approach was used to investigate spatial correlation in the hierarchically arranged datasets. The spatial component of the model decomposes cubicle-level variation into a spatially structured component and a spatially unstructured component, thereby encapsulating the influence of unmeasured predictor variables that themselves are spatially clustered and/or random. A fixed effect for the presence of another patient with the same type of MRSA in the cubicles two weeks prior was included.

**Results:**

The best-fitting model for non-multiresistant MRSA had an unstructured random effect but no spatially structured random effect. The best-fitting model for multiresistant MRSA incorporated both spatially structured and unstructured random effects. While between-cubicle variability in risk of MRSA acquisition within the hospital was significant, there was only weak evidence to suggest that MRSA is spatially clustered. Presence of another patient with the same type of MRSA in the cubicles two weeks prior was a significant predictor of both types of MRSA in all models.

**Conclusions:**

We found weak evidence of clustering of MRSA acquisition within the hospital. The presence of an infected patient in the same cubicle two weeks prior may support the importance of environmental contamination as a source of MRSA transmission.

## Background

Methicillin-resistant *Staphylococcus aureus* (MRSA) is a major antibiotic-resistant pathogen causing healthcare-acquired infections (HAIs) in hospitals worldwide [1]. Transmission of MRSA is through indirect patient contact via the healthcare worker or a contaminated environment [2-5]. Close proximity to a previously colonized or infected patient in the same cubicle is a known risk factor for HAI transmission [6-8]. Environmental transmission routes in the hospital may include fomite contamination [2,5,9,10] and aerosol dispersal [11,12].

Few studies have investigated spatial patterns of MRSA transmission in hospitals. Hospitals have an inherently hierarchical structure, with beds positioned within cubicles and cubicles positioned within wards. Patient proximity may facilitate the transmission of MRSA. Hence, the spatial arrangements of beds, cubicles and wards could be important in understanding MRSA transmission risk. Identifying high-risk areas within the hospital could be useful in the design of more effective, targeted MRSA interventions.

Previous empirical studies of the spatial distribution of infections in hospitals have focused on specific wards [13,14], rather than the whole hospital. Modelling studies have also been conducted at the ward level [15]. Few studies have differentiated between new acquisitions and old colonisations of nosocomial pathogens using empirical data, with a rare example being Kho et al. [14]. This is important because new acquisitions are more likely to reflect HAI transmission patterns within the hospital. The current study evaluates spatial clustering of new MRSA acquisitions in a tertiary hospital and thus presents a unique attempt to explore spatial patterns of MRSA transmission at the scale of a large, comprehensive and complex healthcare institution.

In our recent multi-level modelling study, the risk of MRSA infection increased in patients when another infected patient was located in the same cubicle or ward two weeks previously [16]. However, spatial relationships between cubicles were not incorporated in the model. The omission of spatial autocorrelation (i.e. clustering) from the previous study could have influenced the observed association between presence of a patient with MRS and subsequent infection risk in patients in the same cubicle.

The aims of this study were to determine if MRSA acquisition risk is spatially clustered in the hospital and whether identification of high-risk, neighbouring cubicles could be useful in targeting interventions to detect and prevent MRSA transmission, as well as to obtain statistically robust estimates of the association between prior infections in the cubicle and subsequent infection risk.

## Methods

### Study population

The study was conducted in the Princess Alexandra Hospital (PAH), a 796-bed tertiary hospital in Brisbane, Queensland, Australia, from 1st January 2007 to 30th December 2007 (52 weeks). The study included 211 cubicles of the 25 acute-care wards in the hospital. Data on each bed within a hospital was obtained from the Hospital Based Corporate Information System (HBCIS). MRSA screening data were extracted from the eICAT database (Center for Healthcare Related Infection Surveillance and Prevention (CHRISP), Brisbane, Australia), which is maintained by the Infection Management Services (IMS) of the PAH for surveillance purposes. The intensive care unit (ICU) and the infectious diseases ward performed active surveillance, which requires each patient to be screened upon admission for MRSA and twice weekly thereafter. Other isolates were obtained only from clinical specimens as medically indicated. Approval for the study was obtained from the Princess Alexandra Hospital (PAH) Human Research Ethics Committee (HREC) and the University of Queensland Research and Ethics Committee.

### Definitions used

Healthcare-acquired (HA) MRSA is defined as an infection or colonisation, which is acquired during a hospital stay, and was not present or incubating on admission, and that occurs >48 hours after hospital admission or <48 hours after discharge (CHRISP, 2004). Antimicrobial sensitivity testing methods categorised HA MRSA as non-multiresistant MRSA (nmMRSA), multiresistant MRSA (mMRSA) and UK epidemic-variant MRSA (UKeMRSA) in our study [17]. These categories of MRSA types are not reflective of specific genetic strains. For the purpose of this analysis, UKeMRSA was excluded due to low prevalence within the hospital. New MRSAs were defined in the eICAT database as new isolates from which patient had no previous clinical history of MRSA. Repeated tests on the same patients were excluded. The spatial unit of the study was the hospital cubicle, the definition of which is an enclosed space surrounded by walls. Adjacent cubicles were those with a shared wall. Cubicles included single-bed isolation rooms, shared multi-bed cubicles and open-plan wards (such as the ICU).

### Spatial layout of the hospital

The hospital is organised into levels (i.e. floors), each containing wards that are interconnected, being separated by doorways. An exception is the Spinal Injury Unit (SIU), situated in a separate building. The ICU does not share a common entrance or a patient walkway with other wards. The infectious disease ward and CCU are separated from the common walkways and entrances through security access doors. The infectious disease ward has a negative pressure ventilation system and two secured doors, which separates it from the adjacent general medical ward. The design of both the ICU and coronary care unit (CCU) is open, with no sub-division by walls. In multi-bed cubicles, curtains separate each bed for individual patient privacy.

### Statistical methods

A reference list of the unique record (UR) numbers of all patients with MRSA acquisitions was extracted from the eICAT database. The UR number was used to manually extract information on patient location at the time of their new MRSA acquisition from the HBCIS database. Storage, linkage and management of data from the different sources were done in Microsoft® Access (Microsoft Corporation, Redmond, Washington). A matrix of cubicles (211) by weeks (52, defined by the days Sunday to Saturday) was generated. A variable describing whether or not each bed-week had an acquisition of MRSA was determined (coded as 0 = did not occur; 1 = did occur). This was done separately for nmMRSA and mMRSA. Additionally, a predictor variable (identified in our previous study) was included: if a patient in the same cubicle was identified as being colonised or infected with the same type of MRSA two weeks previously (i.e. during the week prior to the last week). We also tested a new predictor variable: cubicle type (single bed or shared), but this was not significant in preliminary analyses (*P* > 0.2) and was excluded from subsequent models. Finally, an index variable was generated identifying the cubicle in which each bed was located.

Bayesian spatio-temporal modelling approach was chosen because it is appropriate for hierarchically arranged datasets, provides a means for investigating spatial correlation and allows quantification of the uncertainty in the model estimates. For the spatial component of the model, the approach of Besag et al. [18] was chosen, involving the inclusion of spatially structured and unstructured random effects. The spatially structured random effect captures the component of residual variation (after accounting for the predictor variables) that is spatially correlated; and the unstructured random effect captures the spatially random component. These components partly encapsulate the influence of unmeasured predictor variables that themselves are spatially structured or unstructured. In the case of infectious diseases, the spatially structured component also encapsulates the effect of proximity on transmission, which results in the typically clustered spatial pattern of many types of infection. The models were built in a sequential fashion, first with an unstructured random effect, then with a spatially structured random effect, and finally with both structured and unstructured random effects (a so-called “convolution prior” model).

Models were constructed in a Bayesian framework using the software WinBUGS version 1.4 (MRC, Cambridge, UK and Imperial College London, UK). For each type of MRSA (mMRSA and nmMRSA), the dependent variable was a Bernoulli-distributed variable (i.e. 1 = MRSA acquisition, 0 = no MRSA acquisition) and was the probability of MRSA acquisition for the -th bed in the -th cubicle in week *t*. The initial model with the unstructured cubicle-level random effect was:

$$Y_{\mathrm{ijt}}\;∼\;\mathrm{Bernoulli}\left(P_{\mathrm{ijt}}\right)$$

(1) $$\mathrm{Logit}\;P_{\mathrm{ijt}}\;=\;\alpha \;+\;\beta x_{\mathrm{jt}}\;+\;C_{i}\;+\;\mathrm{ST}\;+\;v_{j}$$

Where *α* is the intercept and a Bernoulli-distributed variable, *x*_*jt*_ (with coefficient *β*) described whether or not a patient with MRSA was present in the same cubicle two weeks prior, *C*_*t*_ is a Gaussian-distributed random effect for beds (acknowledging that observations are repeated for each bed over the 52-week period), represents temporal trend by week, where *T* = weeks 1:52, *v*_*j*_ and represents the spatially unstructured cubicle-level random effect.

The second model, with the spatially structured random effect was:

(2) $$\mathrm{Logit}\;P_{\mathrm{ijt}}\;=\;\alpha \;+\;\beta x_{\mathrm{jt}}\;+\;C_{i\;}\mathrm{\delta t}\;+\;u_{j}$$

where *u*_*j*_ is the spatially structured random effect with a conditional autoregressive (CAR) structure. Here, spatial relationships between the cubicles were defined using an adjacency weights matrix. Adjacent cubicles that shared a wall were considered neighbours; weights = 1 for neighbouring cubicles and 0 for non-neighbouring cubicles. The value of each spatially structured random effect was dependent on the mean of the risk and the variance in adjacent cubicles, with information from non-neighbouring cubicles only influencing the random effect estimate of a given cubicle indirectly, through their influence on the neighbours. There was a total 355 neighbouring pairs of cubicles. Given that no pairs of cubicles in different levels of the hospital shared a wall, no cubicles from other levels were neighbours and no information could flow between the levels of the hospital – the levels were, in effect, considered to be islands.

The third model incorporated both the spatially unstructured and structured effects:

(3) $$\begin{array}{ll} \text{Logit}\;P_{\mathrm{ijt}}= & \;\alpha \;+\;\beta x_{\mathrm{jt}}\;+\;C_{i\;}+\mathrm{\delta t}\\ & +\;u_{j}\;+\;v_{j} \end{array}$$

Standard, non-informative prior distributions were specified for the intercept, coefficients and parameters defining the random effects. An unbounded uniform (i.e. flat) prior distribution was specified for the intercept. In WinBUGS, Gaussian distributions are described using the mean and precision, where precision is the inverse of the variance, such that a small value for the precision equates to a large variance. For the model coefficients (*β*), priors were Gaussian distributions with a mean = 0 and precision = 0.0001 (equating to a variance of 10,000, giving a suitably flat probability distribution). Priors for the precision of the bed and cubicle level random effect were specified using gamma distributions with shape and scale parameters (1, 0.1).

Markov-Chain Monte Carlo simulation with Gibbs sampling was implemented in WinBUGS to obtain the posterior distributions of the unknown model parameters. Samples of 150,000 iterations were taken from the posterior distributions of all model parameters following an initial burn-in of 50,000 iterations. To minimize autocorrelation in the Monte Carlo chains, every 25th iteration was used to estimate the posterior distributions. The deviance information criterion (DIC) was compared for the models containing unstructured and spatially structured random effects. A model with a smaller DIC denotes a better fit.

Maps were constructed using ArcMap 10.0 GIS software (ESRI, Redlands, CA) to visualize the risk of MRSA acquisition. The mean of the spatially unstructured and structured random effects in the convolution prior models for each type of MRSA were mapped in the GIS.

## Results

There were 162/29224 (0.55%) bed-weeks in which a patient had a newly acquired nmMRSA, and 416/29224 (1.42%) for mMRSA. The infectious disease ward had the highest number of newly acquired MRSA, including 24.1% of nmMRSA and 14.4% of mMRSA detected in patients. The ICU followed with 17.3% of newly acquired nmMRSA and 12.7% of mMRSA. The spinal injury ward accounted for 10.5% of newly acquired nmMRSA while one of the general medical wards had 11.0% of all mMRSA.

For nmMRSA, the best-fitting model was the one with the unstructured random effect but no spatially structured random effect, while for the mMRSA, the best-fitting model incorporated both the spatially structured and unstructured random effects (the convolution prior model), as seen in Table 1. The unstructured variation was much greater than the spatially structured variation in the convolution models; in other words, most of the variability in MRSA risk was spatially random throughout the hospital.

**Table 1:** Bayesian hierarchical spatio-temporal (two-week lag) models of non-multiresistant and multiresistant methicillin resistant Staphylococcus aureus in a tertiary hospital, Brisbane, Australia, 2007

	**nmMRSA**			**mMRSA**		
	**Unstructured random effects *(v)***	**Spatially structured random effects *(u)***	**Spatially unstructured and structured random effects *(v + u)***	**Unstructured random effects *(v)***	**Spatially structured random effects *(u)***	**Spatially unstructured and structured random effects *(v + u)***
Parameters	Mean (95% CrI )	Mean (95% CrI )	Mean (95% CrI )	Mean (95% CrI )	Mean (95% CrI )	Mean (95% CrI )
Intercept	-6.34 (-6.86 to 5.88)	-6.79 (-7.35 to -6.26)	-9.55 (-11.99 to -7.75)	-4.33 (-4.60 to -4.06)	-4.65 (-5.03 to -4.33)	-8.83 (-8.81 to -7.74)
Odds ratio*	7.09 (4.53 to 11.05)	10.29 (6.83 to 15.10)	6.42 (4.10 to 10.18)	4.15 (3.18 to 5.47)	4.75 (3.72 to 6.12)	6.17 (4.14 to 10.07)
Trend coefficient	1.01 (1 to 1.02)	1.01 (1 to 1.02)	1.02 (1 to 1.02)	0.98 (0.97 to -0.98)	0.98 (0.97 to -0.98)	0.99 (0.97 to -0.99)
Variance Cubicle-level random effect	1.71 (0.98 to 2.73)	0.21 (0.05 to 0.56)	1.49 (0.63 to 2.92)*(v)*; 0.11 (0.02 to 0.36)*(u)*	1.21 (0.78 to 1.71)	0.34 (0.12 to 0.68)	1.18 (1.42 to 2.31)*(v)*; 0.11 (0.02 to 0.36)*(uc*
DIC	**1700.51**	1754.34	1742.86	3819.19	3865.12	**3815.46**

*For presence of a patient with the same type of MRSA in the cubicle two weeks prior.

For nmMRSA, the three cubicles with significantly higher than average risk of new acquisitions were situated in the infectious disease ward, with the respective mean log relative risks (log RR) of 3.13 (95% CrI: 1.88 – 4.21), 2.64 (95% CrI: 1.86 – 3.39) and 2.58 (95% CrI: 1.18 – 3.82) for the unstructured spatial random effect; and 2.87 (95% CrI: 1.89 – 3.89), 2.67 (95% CrI: 1.86 – 3.46) and 2.91 (95% CrI: 2.03 - 3.86) for the structured random effect respectively.

For the mMRSA, the two cubicles with significantly higher than average risk of new acquisitions were situated in the general medical ward and the ICU, with a mean log RR for the unstructured random effect of 2.20 (95% CrI:1.17 – 3.11) and 0.54 (0.14 – 0.93), respectively.

**Figure 1:**
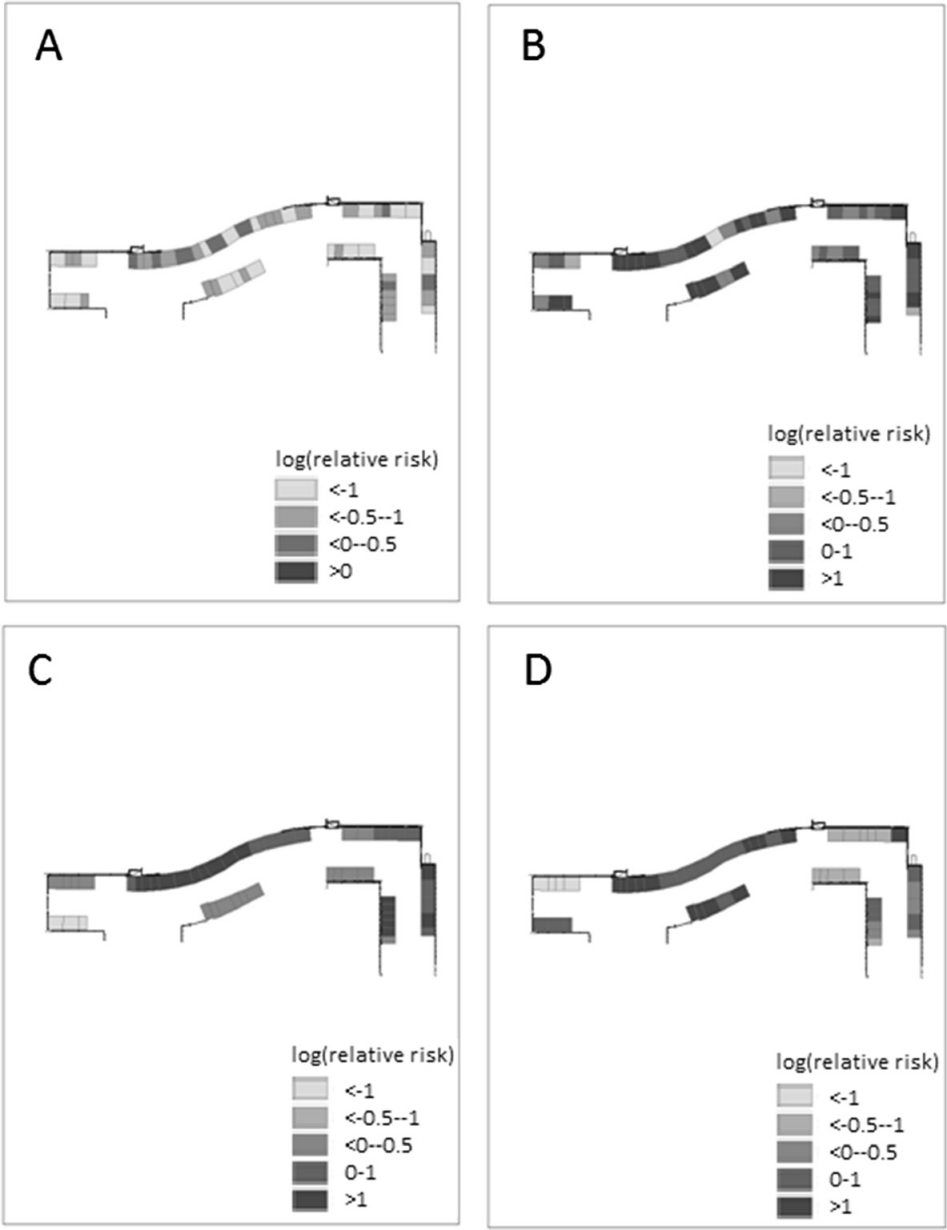
**Maps of mean values (log relative risk) of random effects for MRSA acquisition risk by cubicle in a representative level of a tertiary hospital, Brisbane, Australia, 2007. A**. spatially unstructured random effect, non-multiresistant MRSA. **B**. spatially unstructured random effect, multiresistant MRSA. **C**. spatially structured random effect, non-multiresistant MRSA. **D**. spatially structured random effect, multiresistant MRSA.

A mapped example from one of the floors of the PAH shows the smooth spatial pattern of risk captured by the spatially structured random effects, indicating clustering of MRSA risk at the cubicle level, and the spatially random pattern of risk captured by the unstructured random effects (Figure 1). Note, the scale of the figure is log RR (negative values indicate lower than average risk and positive values indicate higher than average risk) and the results shown are for the convolution prior model for both types of MRSA.

## Discussion

The findings of this study indicate that, although there is significant between-cubicle variability in risk of MRSA acquisition within the hospital, most of the variability of MRSA acquisition risk was spatially random. The highest risk of new nmMRSA acquisition was found in cubicles in the infectious disease ward, which is the main isolation ward of the hospital and is one that carried out active surveillance. The highest risk of new mMRSA acquisition was found in a cubicle of a general medical ward. The infectious disease ward and this particular general medical ward are neighbouring wards and this ward is used as an overflow for infectious disease patients.

Between-cubicle variation in MRSA acquisition risk may be explained by: active surveillance only being done in high risk wards leading to a variable detection rate (with potential underestimation of acquisition risk in wards that do not practice active surveillance); isolation policies within the hospital that group high-risk or infected patients into specific cubicles; assignment of staff (who might have different levels of hand hygiene compliance or other patient care practices) to specific beds or cubicles [19,20]; presence of patients with similar MRSA acquisition risk within the same ward (with similar risk factors, co-morbidities, or invasive devices); and differences in antimicrobial usage amongst the wards [21-24].

Hand hygiene has been the traditional focus of infection control practices in most hospitals. This study confirms our previous study that found that presence of a patient in the same cubicle two weeks prior with the same type of MRSA had a strong association with subsequent MRSA acquisition risk. This suggests that residual environmental contamination is potentially a major driver of MRSA transmission in the hospital. Pathogens, such MRSA, are known to survive for at least 4–5 months in the environment under the right conditions [25]. MRSA can be shed from infected patients on to surfaces, or via mucosal discharges (leading to airborne dispersal). The risk of subsequent infection is dependent on the dosage of infective particles in the environment. Contamination of rooms with viable organisms shed by previous patients could lead to subsequent MRSA colonisation and infection [25-27]. There is some degree of difficulty in objectively measuring environmental cleanliness, perhaps resulting in very few studies which are focused specifically on environmental transmission environmental transmission studies of HAI [4,25]. Dispersal of pathogens by hospital patients through shedding, and bed-making activities in neighbouring beds may explain the fact that proximity to infected patients is a risk factor [10,12,28,29].

Decontamination of the environment can either refer to disinfection and/or cleaning. Disinfection refers to inactivation of pathogens while cleaning refers to the removal of the contaminant from the surface using a detergent. A specific disinfectant may inactivate one pathogen but not another. Ineffective cleaning of equipment may actually spread the active pathogen on to other surfaces [4,25]. Despite routine cleaning and hand-washing efforts, equipment contamination may still occur, promoting pathogen transmission directly and indirectly through staff and patient contact with affected surfaces [4,30]. Patients may have direct contact with contaminated surfaces from a previous infected occupant in the room or bed, while staff may have unknowingly used inadequately cleaned/disinfected or contaminated products or equipment within the ward, transmitting organisms to susceptible patients [4,26,27,30]. Shared access to decontamination areas and 'high touch’ equipment (i.e. intravenous pumps, bed rails, bedside tables, keyboards at nurse’s stations) could lead to transfer of pathogens from staff looking after infected or colonised patients to other staff without direct exposure to an infected patient [4,26,31]. Starr et al. [32] suggested that environmental cleaning data together with information on staff behaviour may be useful to ascertain the risk of HAI transmission and evaluate infection control measures in future studies.

The only predictor variables considered in this analysis were presence of an infected patient in the same cubicle in preceding weeks, and whether the cubicle was a single or multi-bed cubicle. Future investigation should include other predictor variables that capture patient case-mix and ward activity levels [33-35]. We only used the type of MRSA (nmMRSA or mMRSA) to indicate risk of infection associated with infection or colonisation of a previous patient, rather than genotyping. Our study was, therefore, limited in its ability to identify specific transmission events. Future molecular epidemiological studies will help illuminate the transmission dynamics of MRSA and other HAI, including spatial patterns in transmission.

Due to the predominance of spatially random variation in risk of new nmMRSA and mMRSA acquisitions, routine targeting of high-risk cubicles or wards within the hospital for specific interventions may not be possible. Approaches to infection control should be applied throughout the hospital and, in the event of nmMRSA or mMRSA transmission being identified in a particular cubicle, infection control interventions can be monitored with more rigorous enforcement, to reduce risk of MRSA acquisitions in other patients in the following two-week period.

## Conclusions

The degree of spatial clustering of MRSA transmission across neighbouring cubicles in a multi-level hospital was found to be low in this study. Environmental cleaning and disinfection may need to be emphasised further given the significant finding that patients are at a higher risk of MRSA acquisitions two weeks after a MRSA infection or colonisation is identified. Specific targeting of cubicles for enhanced cleaning and disinfection in this period may be warranted to reduce the new MRSA acquisitions.

## Competing interests

The authors declare that they have no competing interests.

## Authors’ contributions

FK did the analysis and drafted the manuscript. DLP, MW and MC provided advice on the clinical significance of the research findings and contributed to drafts of the manuscript. ACAC provided guidance on study conception, data analysis and drafting of the manuscript. All authors read and approved the final manuscript.

## Pre-publication history

The pre-publication history for this paper can be accessed here:

http://www.biomedcentral.com/1471-2334/13/449/prepub
